# Evaluation of the Efficiency of Ethanol Precipitation and Ultrafiltration on the Purification and Characteristics of Exopolysaccharides Produced by Three Lactic Acid Bacteria

**DOI:** 10.1155/2018/1896240

**Published:** 2018-09-18

**Authors:** Manel Ziadi, Taroub Bouzaiene, Sana M'Hir, Kaouther Zaafouri, Ferid Mokhtar, Mokhtar Hamdi, Claire Boisset-Helbert

**Affiliations:** ^1^Laboratory of Microbial Ecology and Technology, LETMi-INSAT, National Institute of Applied Sciences and Technology INSAT, Carthage University, 2 Boulevard de la Terre, BP 676, 1080 Tunis, Tunisia; ^2^Centre de Recherche sur les Macromolécules Végétales, CERMAV, CNRS, 601 rue de la Chimie, 38041 Grenoble Cedex 9, France; ^3^National Research Center for Materials Science, Borj-Cedria Technopark, BP N°73, 8027 Soliman, Tunisia

## Abstract

Exopolysaccharides (EPS) produced by three Lactic Acid Bacteria strains,* Lactococcus lactis* SLT10,* Lactobacillus plantarum* C7, and* Leuconostoc mesenteroides* B3, were isolated using two methods: ethanol precipitation (EPS-ETOH) and ultrafiltration (EPS-UF) through a 10 KDa cut-off membrane. EPS recovery by ultrafiltration was higher than ethanol precipitation for* Lactococcus lactis* SLT10 and* Lactobacillus plantarum* C7. However, it was similar with both methods for* Leuconostoc mesenteroides* B3. The monomer composition of the EPS fractions revealed differences in structures and molar ratios between the two studied methods. EPS isolated from* Lactococcus lactis* SLT10 are composed of glucose and mannose for EPS-ETOH against glucose, mannose, and rhamnose for EPS-UF. EPS extracted from* Lactobacillus plantarum* C7 and* Leuconostoc mesenteroides* B3 showed similar composition (glucose and mannose) but different molar ratios. The molecular weights of the different EPS fractions ranged from 11.6±1.83 to 62.4±2.94 kDa. Molecular weights of EPS-ETOH fractions were higher than those of EPS-UF fractions. Fourier transform infrared (FTIR) analysis revealed a similarity in the distribution of the functional groups (O-H, C-H, C=O, -COO, and C-O-C) between the EPS isolated from the three strains.

## 1. Introduction

Exopolysaccharides (EPS) are long-chain polymers, industrially used as thickeners, stabilizers, and gelling agents in food products. More recently they were used as depollution agents and there was a growing interest in their biological functions like antitumor, antioxidant, or prebiotic activities [1]. Exopolysaccharides are produced by the metabolic processes of microorganisms such as bacteria, fungi, and blue-green algae [2]. Bacterial Exopolysaccharides are widely described in the literature, offering a wide range of biological and physicochemical properties.

Lactic Acid Bacteria (LAB) represent a natural source of EPS which play an important role in the rheological behavior and texture of fermented milks [3–5]. Most LAB producing EPS belong to the genera* Streptococcus*,* Lactobacillus, Lactococcus, Leuconostoc*, and* Pediococcus *[6].

EPSs from LAB can be classified into two groups: homopolysaccharides and heteropolysaccharides. Homopolysaccharides consist of repeating units of only one type of monosaccharide (D-glucose or D-fructose) and can be divided into two major groups: glucans and fructans. By contrast, heteropolysaccharides, produced by a great variety of mesophilic and thermophilic LAB, are formed by repeating units that most often contain a combination of D-glucose, D-galactose, and L-rhamnose and, in a few cases, N-acetylglucosamine (GlcNAc), N-acetylgalactosamine (GalNAc), or glucuronic acid (GlcA). Sometimes, noncarbohydrate substituent such as phosphate, acetyl, and glycerol are present. The molecular mass of these polymers ranges between 40 and 6000 kDa [4]. Heteropolysaccharides from LAB demonstrate different structures [7]. The heteropolysaccharides are constructed from multiple copies of oligosaccharides, which contain between three and eight residues. Two or more different monosaccharides are usually present in each repeating unit and show different linkage patterns [8].

EPSs produced by LAB are in great variety, depending on LAB strains, culture conditions, and medium composition [9], and often differ by monosaccharides composition, charge, linkages between units, and presence of repeated side chains. The sugar components of EPS from LAB are most commonly galactose, glucose, and rhamnose [3]. The EPSs isolated from some strains of* Lc. lactis* subsp.* cremoris* contain rhamnose, glucose, galactose, and phosphates [10–12], while others contain only glucose and galactose. Marshall et al. [13] found that* Lc. lactis* subsp.* cremoris* LC330 produced two EPSs with different sugar composition and molecular mass: a neutral EPS of 1.106 kDa and a smaller negatively charged EPS (containing phosphate groups) of about 1.104 kDa. Van Casteren et al. [14] reported that EPS from* Lc. lactis* subsp.* cremoris* B40 consists of rhamnose, galactose, and glucose in the ratio of 0.9:1.2:2.0 and that the molar ratio of carbohydrate and phosphorus is 4.7:1.* Streptococcus thermophilus* produce an EPS composed of galactose and rhamnose when grown on milk [9].* Lactobacillus bulgaricus *grown on chemically defined medium produce an EPS composed of galactose, glucose, and rhamnose [15]. Many strains of* Leuconostoc mesenteroides* produce dextran (*α*-glucan). Levans are produced by several strains of* Streptococcus mutans* [3].

Wide range of exopolysaccharides extraction, purification, and analysis schemes have been developed in literature involving from simple dialysis against water of the culture medium following by freeze drying to size exclusion column for preparing of highly pure EPS extracts.

Some authors used trichloroacetic acid (TCA) for protein sedimentation, dialysis for final EPS purification from sugars, or just numerous precipitations with ethanol and/or acetone [6]. Others procedures have been used for EPS purification including microfiltration, ultrafiltration, and diafiltration which can be carried out separately or in combination with ethanol precipitation [16–18]. Different types of membranes have been used such as regenerated cellulose and polyethersulfone, as well as different molecular weight cut-off [19].

In order to obtain pure polysaccharide fractions, size exclusion chromatography is the most common method used because it allows the separation of polysaccharides according to their size and also permits the subsequent determination of their molecular weight [19].

These different methods can be compared according to two criteria: quantity and quality of extracted EPS. It was shown that different extraction procedures influence the quantity and the composition of the extracted EPS [20, 21], the quantity and the composition of the mineral fraction present in the EPS extracts [22], and EPS binding properties to protons and different metals [20, 23, 24].

More advanced technologies to obtain polysaccharides have been used recently, as ultrasonic [25] and microwave assisted extractions [26] besides the pressurized solvent extraction [27]. The latter procedure showed to be faster and more efficient in obtaining higher yield of polysaccharides, comparing to the traditional methodologies.

In the present study, we characterized the EPS fractions obtained from pure bacterial culture of* Lactococcus lactis* subsp.* lactis* var.* diacetylactis* SLT10 (*Lc. lactis* SLT10),* Lactobacillus plantarum* C7 (*Lb. plantarum *C7), and* Leuconostoc mesenteroides* B3 (*Ln. mesenteroides* B3) to evaluate the influence of purification methods on the EPS yield, composition, and molecular weight. The studied purification methods were ethanol precipitation and ultrafiltration.

## 2. Material and Methods

### 2.1. Bacterial Strains

The three studied strains were obtained from the strain collection of Laboratory of Microbial Ecology and Technology (LETMi):* Lactococcus lactis* subsp.* lactis *SLT10 strain had been previously isolated in our laboratory from Tunisian traditional fermented milk [28].* Lactobacillus plantarum* C7 (*Lb. plantarum *C7) had been isolated from gastrointestinal tract of chickens [29] and* Leuconostoc mesenteroides* B3 (*Ln. mesenteroides* B3) had been isolated from Tunisian palm sap [30]. Stock cultures grown in MRS broth were stored at −18°C in 50% glycerol until use.

### 2.2. Fermentation

The MRS-sucrose medium was used for EPS production and contain: 4% (w/v) sucrose, 10 g/L tryptone, 10 g/L meat extract, 5 g/L yeast extract, 5 g/L sodium acetate, 2 g/L disodium phosphate, 2 g/L tri-ammonium citrate, 0.1 g/L MgSO_4_, and 0.05 g/L MnSO_4_ (pH 6.5) [31, 32]. The medium was autoclaved at 121°C for 15 min. The fermentation temperature, inoculum size, and fermentation time were 30°C, 3.0% (v/v), and 24 h, respectively. After incubation, bacterial cells were separated from the EPS preparation by centrifugation (5,000 rpm for 10 min at 4°C) of the culture broth. The supernatant, containing the EPS fraction, was filtered under vacuum through Sartorius cellulose nitrate filters of (0.45 *μ*m pore size) to eliminate cells and large cellular. The obtained supernatant was divided into two batches for the purification step.

### 2.3. Purification of Exopolysaccharides

The obtained supernatant was divided in two batches. The first supernatant was treated with NaCl to a final concentration of 1 M. EPS was precipitated by the addition of v/v chilled ethanol (96%) to the supernatant (EPS-ETOH). The proportion of chilled ethanol (v/v) was chosen in order to precipitate high-molecular weight exopolysaccharides. After precipitation at 4°C (overnight) the sample was centrifuged at 6,000 rpm for 20 min at 4°C, and the pellet was washed with ethanol (96%) and recentrifuged at 6,000 rpm for 20 min at 4°C. The pellet obtained was redissolved in distilled water and lyophilized.

The second supernatant was ultrafiltrated (UF) using a 10 kDa cut-off cellulose membrane. Finally, the exopolysaccharide fraction (EPS-UF), collected from the retentate, was evaporated and lyophilized.

### 2.4. Exopolysaccharides Characterization

#### 2.4.1. Monosaccharide Analysis

The monomer composition of exopolysaccharides extracted from the three studied strains was determined after preparation of methyl glycosides trimethylsylil derivatives. Suspensions were prepared by dissolving 4 mg of lyophilized polysaccharides in 2 mL distilled water. Fifty *μ*L of* myo*-inositol, used as internal standard, was added to 200 *μ*L of polysaccharides suspension. The mixture was hydrolyzed for 4 h at 100°C, in screw glass tube, using 500 *μ*L of methanol/HCl (3 N) (Supelco). After cooling to room temperature, methanolyzate fractions were neutralized with silver carbonate, centrifuged at 6,000 rpm for 5 min, and evaporated under nitrogen flow. The generated methyl glycosides were then converted to their corresponding volatile trimethylsilyl derivatives. The reaction took place by adding 70 *μ*L pyridine and 70 *μ*L derivatization reagent, Bis(tri-methylsilyl) trifluoroacetamide (BSTFA) + 1% trimethylchlorosilane (TMCS) (Supelco), incubated for 30 min at 80°C. After solvent evaporation under nitrogen flow, the generated per-O-trimethylsilylmethyl glycosides were resuspended in 700 *μ*L dichloromethane and analyzed by gas chromatography-flame ionization detector (GC-FID). An Agilent GC 6850A instrument equipped with HP-5MS capillary column (30 m length, 0.25 mm diameter, and 0.25 *μ*m film thicknesses) was used. The GC oven temperature was set to 120°C, increased first to 180°C at 3°C/min, then increased to 200°C at 2°C/min, and held for 5 min. The helium carrier gas flow was set at 1.5 mL/min and the injection volume was 0.1 *μ*L.

#### 2.4.2. Molecular Mass Determination

The average molecular mass of exopolysaccharides extracted from the three studied strains was determined using high-performance size exclusion chromatography (HPSEC) equipped with Shodex OHpak SB-804 HQ and SB-805 HQ columns placed in series. The EPS was eluted with 0.1 M Sodium Nitrate (NaNO_3_) at a flow rate of 0.5 mL.min^−1^. Detection was performed using a refractive index detector (RI) (Waters) and a multiangle laser-light scattering detector (LS) (MiniDAWN, Wyatt Technology, Dawn).

#### 2.4.3. Fourier Transform Infrared (FTIR) Spectroscopy of Purified EPS

The structural characterization of the purified EPS was studied using Fourier transform infrared (FTIR) spectroscopy in order to determine their functional groups distribution. The translucent pellets (5mmØ) were made by mixing and pressing the freeze-dried polysaccharides samples with KBr powder (5:100 w/w). The FTIR spectra were recorded in transmittance mode at a spectral range of 4000 and 400 cm^−1^ with an accumulation of 15 scans and a resolution was 4 cm^−1^, using a spectrophotometer type Perkin Elmer Spectrum BX®, equipped with a He-Ne laser and a detector MCT type broadband and high sensitivity [33]. The spectra acquisition was performed via spectrum v5.3.1 software. The bands identification was accomplished according to the data cited by Wiercigroch et al. [34].

### 2.5. Analytical Methods

#### 2.5.1. Total Sugar Determination

Total carbohydrate content of the exopolysaccharides obtained by ethanol precipitation and ultrafiltration was estimated by the phenol-sulfuric acid calorimetric method [35]. A 200 *μ*l EPS sample solution (10 mg.mL^−1^) was treated with 200 *μ*l of a 5% phenol solution and 1 ml of pure H_2_SO_4_. The mixture was cooled to room temperature for 30 min. Absorbance at 492 nm of samples as well as standard sugar (10 to 100 *μ*g.mL^−1^ ribose) was read by spectrophotometry (Hitachi U-1800®).

#### 2.5.2. Protein Concentration Measurement

Protein content was calculated according to the Bradford's method [36]. 1 mL of reagent (Biorad) was added to 20 *μ*L of sample (10 mg.mL^−1^) and incubated 5 min at room temperature. OD of samples as well as the standard protein (0 to 0.75 mg.mL^−^1 of Bovine Serum Albumin (BSA)) was measured at 595 nm (Hitachi U-1800®).

### 2.6. Statistical Analysis

All experiments were performed in triplicate and are reported as means ± standard deviation. Significant differences between samples were tested using a two-sample comparison analysis and a* t*-test. The statistical significance of the relationship was analyzed at the 95% confidence level.

## 3. Results and Discussion

The three studied strains were isolated from different ecological niches.* Lc. lactis *SLT10 is a starter used for the preparation of “Leben” a Tunisian fermented milk;* Lb. plantarum *C7 is a probiotic strain isolated from gastrointestinal tract of chickens. For both strains, characterization of EPS produced can provide important data concerning their uses as starters for the preparation of fermented functional foods. As regards* Ln. mesenteroides* B3, this is the first time that a strain isolated from Tunisian palm sap was investigated for EPS production.

### 3.1. Effect of Purification Method on Exopolysaccharides Yield

In order to isolate EPS produced by the three studied strains, two purification methods were used: ethanol precipitation and ultrafiltration (UF) through 10 kDa membrane. Molar exclusion limit of 10 kDa has been chosen based on preliminary study showing that bacterial polysaccharides fractions presented molecular weight higher than 10 kDa. Moreover, with molecular weight cut-off of 10 kDa high- and low-molecular weight, polysaccharides are retained, while oligosaccharides, polypeptides, and so forth are removed.

The evaluation of the efficiency of both methods on polysaccharides recovery after purification step was conducted using the phenol-sulfuric method described by Dubois et al. [35] for the determination of sugars and related compounds on the freeze-dried extract. This method has been widely used as an indication of the EPS yield after different purification methods. Results are summarized in Table 1.

As shown in Table 1, EPS recovery by UF was significantly higher than ethanol precipitation for* Lc. lactis* SLT10 and* Lb. plantarum* C7 (t<0.05). UF was more efficient than ethanol precipitation method (1.2 times more). However, EPS recovery was similar for both methods for* Ln. mesenteroides* B3. Indeed, UF is widely used method for polysaccharides purification. Similar results were found by Bergmaier et al. [16], who compared UF with a conventional method based on ethanol precipitation, dialysis, and protein removal by trichloroacetic acid; the EPS recovery by the UF was higher.

Higher EPS recovery (70.38%) was, also, found by Pan and Mei [32] when using UF for the purification of EPS obtained from* Lc. lactis *subsp*. lactis* 12. Tuinier el al. [17] used UF with a polysulfone membrane (molar exclusion limit10 kDa) after a microfiltration step for the separation of EPSB40 from* Lc. lactis* subsp.* cremoris* on whey based media; the freeze-dried extract contains 63% EPS.

Polysaccharides yields obtained in this work (Table 1) were low compared to literature. This result can be explained by the following: (i) both methods used are not appropriate to isolate EPS which affects strongly the final yield; (ii) EPS production by the three studied strains was limited in these culture conditions. Remada and Abraham [37] studied the effect of a heat treatment of the milk on EPS recovery and found that the highest recovery of EPS was obtained when samples were heated as a first step of isolation. The heat treatment allows the separation of polysaccharide attached to cells since that it has been shown that LAB express at least two distinct phenotypic forms of EPS, either ropy and/or capsular forms [38]. In the procedures without heat treatment, part of the polysaccharide attached to cells would be lost with pellet during broth culture centrifugation. Moreover, heat treatment inactivates the enzymes that could hydrolyze the polymer (glycohydrolases). Remada and Abraham [37] suggested also a method involving 1 or 2 steps of ethanol precipitation followed by dialysis with different cut-off membranes than TCA precipitation.

Proteins content of freeze-dried extracts for both methods was evaluated using Bradford's assay. Results are shown in Table 2.

The low proteins content (about 1%) of all studied samples approves the efficiency of these methods to separate proteins from polysaccharides and to provide a high purity extracts. Similar results (1% of proteins) were found by Maalej et al. [39] for EPS22 extracted from* Pseudomonas stutzeri* AS22 by applying ultrafiltration followed by dialysis. Pan and Mei [32] indicated an absence of proteins and nucleic acid in the EPS-I extracted from* Lc. lactis *subsp.* lactis* 12 by ultrafiltration. Similar results were also found after the analysis of purified EPS sample from* Lb. plantarum *YW11 using anion-exchange chromatography on DEAE-cellulose with 1.38±0.25% proteins content [40]. The proteins content of EPS from* Leuconostoc* sp. CFR 2181 extracted by Ice-cold isopropyl alcohol precipitation and washed with acetone was only 0.8% [41]. Tuinier el al. [17] obtained proteins content of 18% for* Lc. lactis *subsp*. cremoris* when grown on whey based media.

### 3.2. Monomer Composition and Molar Ratio of Exopolysaccharides

Different methods are available for determining the monomer composition of EPS samples. Methanolysis and per-trimethylsilylation provide samples that can be analyzed by GC. Hanko and Rohrer [42] proposed a simple method, requiring acid hydrolysis followed by monomer detection using high-pressure anion-exchange chromatography with pulsed amperometric detection.

In our work, monosaccharides composition of different polysaccharides fractions were determined according to Kamerling method [43] modified by par Montreuil [44]. Identification and quantification of monosaccharides began with a methanolysis of the polymer. The glycosidic residues are converted to their corresponding volatile trimethylsilyl derivatives and then analyzed by gas chromatography-flame ionization detector (GC-FID). The sugar composition and molar ratios of EPS-ETOH and EPS-UF extracts from the three studied strains are summarized in Table 3.

For SLT10, the main sugars were glucose (Glu) and mannose (Man) for EPS-ETOH in the molar ratio of 0.47:1, while (Glu), mannose (Man), and rhamnose (Rha) were present in EPS-UF in the molar ratio of 0.58:1:0.18. This result suggests that the strain produces heteropolysaccharides. Pan and Mei [32] showed that EPS-I extracted from* L. lactis* subsp.* lactis* 12 is mainly composed of fructose and rhamnose. According to literature,* Lc. lactis* subsp.* lactis* and* Lc. lactis* subsp.* cremoris* produce heteropolysaccharides.* Lc. lactis* subsp.* cremoris* B40 and* Lc. lactis* subsp.* cremoris* SBT 0495 produce an EPS composed of glucose, galactose, rhamnose, and phosphate at a ratio of 2:2:1:1 [11, 14]. This diversity in EPS composition produced by* Lc. lactis* can be explained by genetic studies. Indeed, genes implicated in EPS production in* Lc. lactis* are encoded by plasmid DNA. The first lactococcal eps locus identified was that of* Lc. lactis* subsp.* cremoris* NIZO B40; it comprises 14 plasmid-encoded genes [45]. Since then, partial sequences of eps gene clusters have been identified in* Lc. lactis* subsp. cremoris NIZO B891 and NIZO B35 strains [46]. Cluster consisting of 23 putative EPS biosynthetic determinants has been identified on plasmid pCI658 in* Lc. lactis* subsp.* cremoris* HO2 [47].

GC-MS analysis of the monosaccharide composition of the EPS produced by* Lb. plantarum* C7 showed that the EPS was composed of glucose and mannose in a molar ratio of 0.607:1 for EPS-ETOH and 4.07:1 for EPS-UF, which suggested that the strain produces heteropolysaccharides. Different monomers composition and proportion of* Lb. plantarum *strains are available in the literature. The EPS purified from* Lb. plantarum* YW11 using anion-exchange chromatography is composed of glucose and galactose in a molar ratio of 2.71:1 [40]. EPS extracted from* Lb. plantarum* KF5 presented a monomer composition of mannose, glucose, and galactose in a molar ratio of 1:4.99:6.90 [48]. Li et al. [49] showed that the strain* Lb. plantarum* 70810 isolated from Chinese paocai produced two types of EPS with a monomer composition of glucose, mannose, and galactose in a molar ratio of 18.21:78.76:3.03 and 12.92:30.89:56.19, respectively. Indeed, the monosaccharide composition of EPS produced by LAB can be affected by the type of strains; Laws et al. [8] revealed that the genes coding for EPS synthesis are of plasmid origin in the mesophilic LAB strains (e.g.,* Lactococcus*), but they are chromosomally based in the thermophilic strains (*Streptococcus* and* Lactobacilli*). In general, the EPS-producing ability of LAB is regarded as being unstable. For mesophilic LAB strains, the unstable nature of EPS synthesis is consistent with the genes for EPS synthesis being plasmid bound. For the thermophilic LAB strains, it has been proposed that the loss of EPS-producing character is due to deletions and rearrangement resulting from genetic instability.

For* Ln. mesenteroides* B3, GC analysis showed a monomeric composition of glucose and mannose in a molar ratio of 0.61:1 for EPS-ETOH and 3.48:1 for EPS-UF. Even though, Leuconostocs are well known for the production of homopolysaccharides such as alternan, dextran, and levan from sucrose metabolism [50], the strain* Leuconostoc mesenteroides* B3 produces a heteropolysaccharide composed of glucose and mannose. Indeed, Welman and Maddox [51] revealed that themesophilic LAB such as* Leuconostoc mesenteroides* also produces heteropolysaccharides. Previous study showed the ability of Leuconostocs to produce heteropolysaccharides; the EPS isolated from* Leuconostoc* sp. CFR 2181 consisted mainly of glucose (91%) with minor quantities of rhamnose and arabinose [41].

Based on Table 3, the main observation was the difference in monomer composition of EPS extracted by ultrafiltration and ethanol precipitation for* Lc. lactis* SLT10. Moreover, for the strains* Lb. plantarum* C7 and* Ln. mesenteroides* B3 when comparing both purification methods and in spite of similarity in monomer composition, the molar ratios were different.

This result can be explained by the hypothesis that when sucrose was used as carbon source, the studied strains synthesize mixtures of EPSs. The EPSs can have different structures (different monomeric composition) in the case of the strain SLT10 or different molar ratios with the same monomers for* Lb. plantarum* C7 and* Ln. mesenteroides* B3. Thus, we can suggest that the strain SLT10 produces at least two types of exopolysaccharides with different molecular weight and different monomeric composition: the first one with the higher molecular weight is composed of glucose and mannose and the second one with lower molecular weight probably contains rhamnose. When ultrafiltration is used as purification method, with a 10 kDa cut-off membrane that could retain either high- and low-molecular weight polysaccharides, both EPSs were retained. However for ethanol precipitation only one EPS was precipitated; probably the one with the higher molecular weight composed of glucose and mannose.

For the strains* Lb. plantarum* C7 and* Ln. mesenteroides* B3, there is also the possibility of recovering EPS samples that have identical structure (glucose and mannose) but different molecular masses. In previous studies Comte et al. [20] have showed that the EPS characteristics present qualitative and quantitative differences depending on the method used. They found that the extraction methods using chemical reagents strongly affected the HPSEC (high-pressure size exclusion chromatography) fingerprints of EPS, whereas the physical methods influenced only molecular weight distribution but not HPSEC fingerprints. It had been shown that* Lactobacillus* spp. G-77 produces two homopolysaccharides with different structures [52] (Duenas-Chasco et al., 1998). Degeest and de Vuyst [53] reported the production of a high-molecular mass and a low-molecular mass EPS by* Streptococcus thermophilus* LY03. The production of two polysaccharides by* Lb. rhamnosus* has been reported [54]. A strain of* Lb. reuteri* LB 121 is able to produce two types of homopolysaccharides mainly composed of D-glucose or D-fructose [55].

### 3.3. Molecular Mass Estimation

**Figure 1:**
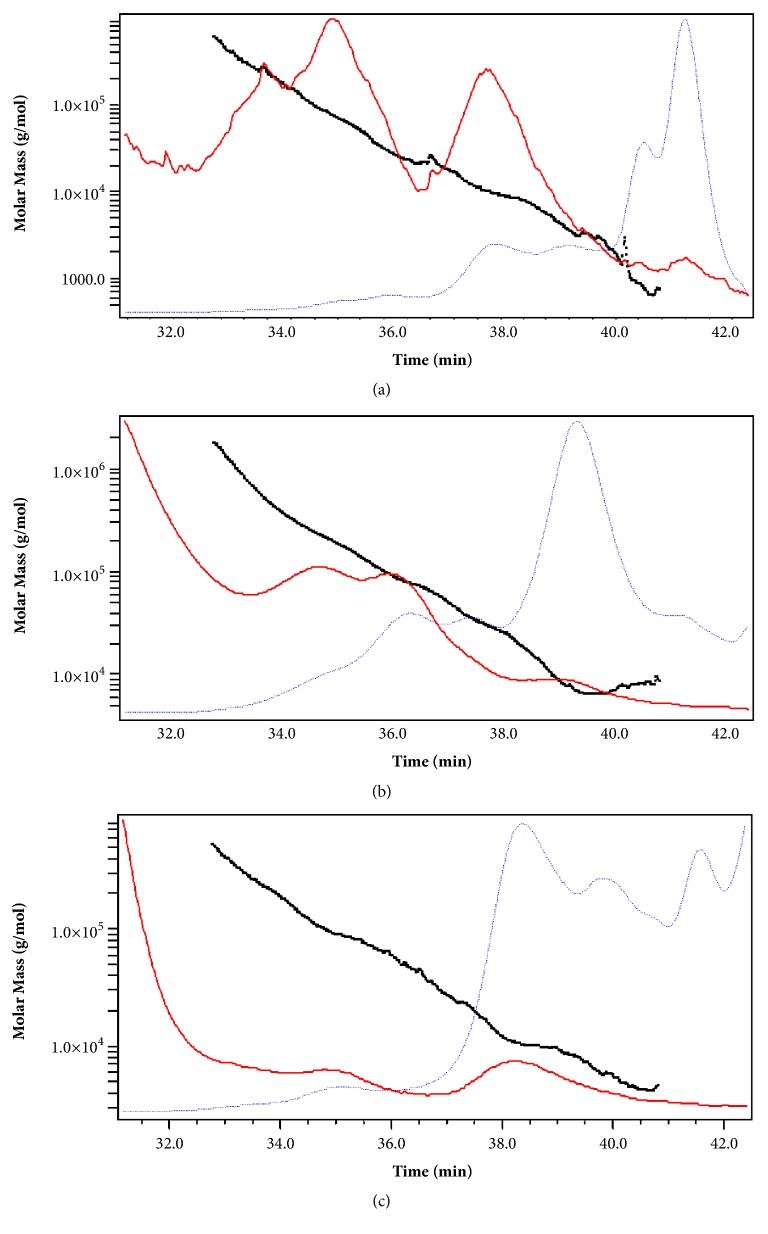
The molecular weight distribution of EPS-UF fractions of* Lb. plantarum *C7 (**a**),* Lc. lactis *SLT10 (**b**), and* Ln. mesenteroides* B3 (**c**) (black squares: molar mass; red dashed line: LS (laser-light scattering); blue dotted line: RI (refractive index)).

**Figure 2:**
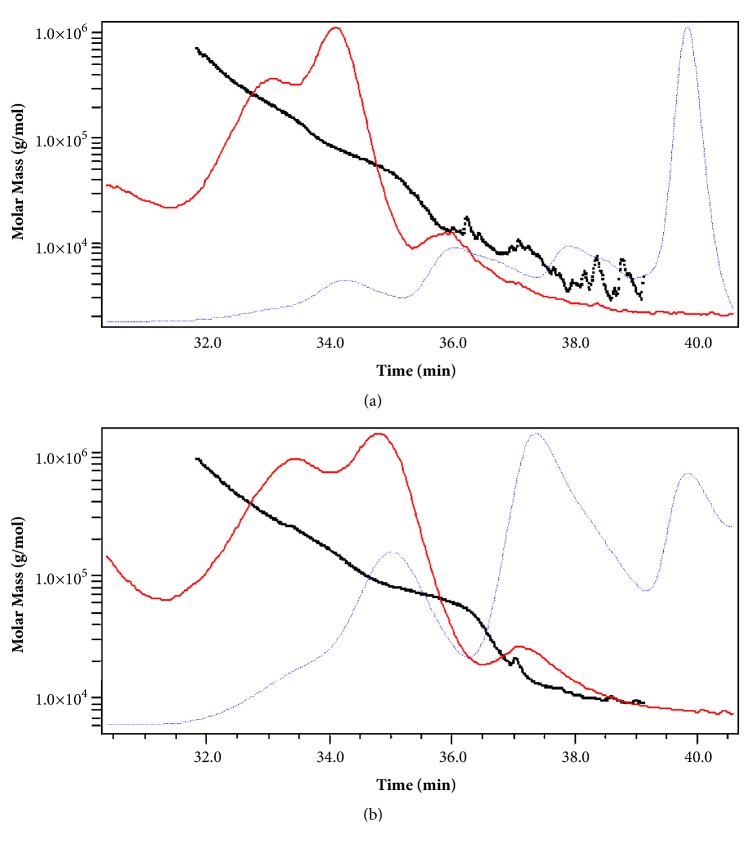
The molecular weight distribution of EPS-ETOH fractions of* Lb. plantarum *C7 (**a**) and* Lc. lactis *SLT10 (**b**) (black squares: molar mass; red dashed line: LS (laser-light scattering); and blue dotted line: RI (refractive index)).

The molecular mass of different EPSs isolated from the three studied strains with both methods was estimated using high-performance size exclusion chromatography (HPSEC). The molecular weight distribution of EPS-UF and EPS-ETOH fractions of* Lb. plantarum *C7,* Lc. lactis *SLT10, and* Ln. mesenteroides* B3 is presented in Figures 1 and 2.

Molecular weight (Mw), molecular number (Mn), and polydispersity index of EPSs isolated from the three studied stains are summarized in Table 4.

The result showed that the three strains present different EPS molecular masses. Indeed, the molar mass of the EPS produced by LAB varied according to strains and polymer type [6]. Generally, the EPSs produced by the bacterial strains in MRS-sucrose media are lower-molecular-mass fractions, whose molecular masses do not exceed 62.4±2.94 kDa. The MW of EPS extracted from* Lc. lactis* SLT 10 was the highest with both purification methods (62.4±2.94 kDa and 51.5 kDa±1.75). Heteropolysaccharides ranged from 40 to 9000 kDa for* Streptococcus thermophilus *strains and 100 to 2000 kDa for* Lc. lactis *spp.* cremoris *strains [6].

The molecular mass of the EPS produced by* Lb. plantarum C7* was determined to be 33.5±2.89 kDa (EPS-ETOH) and 11.6±1.83 kDa (EPS-UF) which was similar to that (44 kDa) of the EPS of* L. plantarum *EP56 [56] but lower than that (110 kDa) of the EPS of* Lactobacillus plantarum *YW11 [40] and (1150 kDa) of the EPS of* Lb. plantarum *C88 [57].

The molecular mass of EPS purified from* Ln. mesenteroides* B3 was only available for EPS-UF and was 18.6±2.21 kDa which in the same range of magnitude of those reported for the EPS fractions of* Leuconostoc* sp. CFR 2181 with molecular weights ranging from 10 kDa to 1500 kDa [41].

The polydispersity index Mw/Mn (Mw, weight-average; Mn, number-average), which reflects the degree of heterogeneity of the polymer's chain lengths, is ranging from 2.204±0.407 to 5.586±2.66 (>1) which represents a heterogeneous populations in terms of polysaccharide chains size. The lower polydispersity index value was obtained for the EPS fraction purified from* Ln. mesenteroides* B3 (2.204±0.407) which can be considered as moderately polydisperse distribution type even when the EPS fractions purified from* Lc. lactis* SLT10 and* Lb. plantarum* C7 were considered as broadly polydisperse distribution type.

The results from Table 4 show two mains observations: (i) EPS-ETOH fractions molecular weights were higher than those of EPS-UF fractions and (ii) the heterogeneity of EPS fractions is more pronounced when UF was used as purification method (polydispersity index ranged from 4.178±0.49 to 5.586±2.66).

These observations can be explained by the hypothesis given above that the studied strains grown in MRS-sucrose produce more than one polymer with various molecular masses causing the heterogeneity of the freeze-dried extracts (high polydispersity index). This hypothesis could explain variations in monomer composition and molecular ratios of EPS-ETOH and EPS-UF. When using UF as purification methods, polymers produced by the lactic strains remain in the retentate. However, when using ethanol for purification, only one polymer precipitates (probably the one with the highest molecular weight). Grobben et al. [15] found that* Lb. bulgaricus* strain NCFB 2772, grown in chemically defined medium, produced two EPS fractions with molecular masses of 40 and 1700 kDa. Similar results were reported with* Lc. Lactis* subsp.* cremoris* LC 330 [13].

### 3.4. The Structural Characterization of the Purified EPS

**Figure 3:**
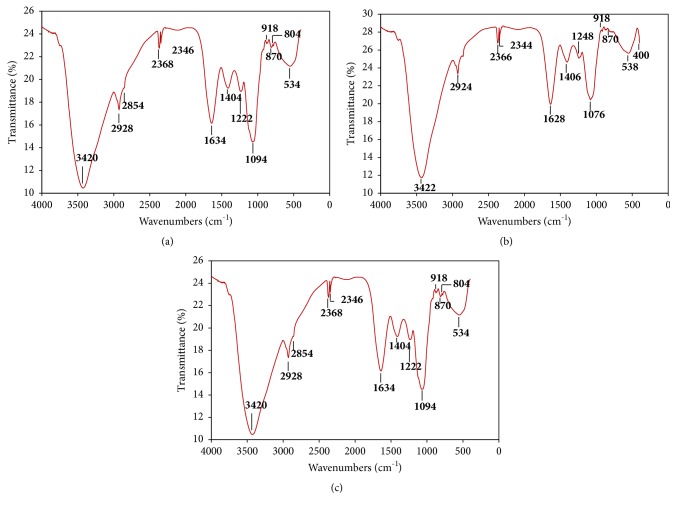
FTIR spectra of EPS-ETOH fractions of* Lb. plantarum* C7 (**a**),* Lc. lactis* SLT10, (**b**) and* Ln. mesenteroides* B3 (**c**).

FTIR spectroscopy has been a powerful and valuable analytical method to investigate the nature of the functional groups of EPS in terms of monomeric units and their linkages.  Figure 3 shows the FTIR spectra of EPS fractions from* Lb. plantarum *C7,* Lc. lactis* SLT10, and* Ln. mesenteroides* B3 obtained by precipitation in a final ethanol concentration 96%.

The spectrum of purified EPS was studied in the region between 400 cm^−1^ and 4000 cm^−1^ and showed numerous peaks from 3434 cm^−1^ to 534 cm^−1^. In comparison with the IR spectra of polysaccharides listed in the literature, all the peaks obtained were in agreement with the typical absorption peaks of polysaccharides.

As can be seen, no significant difference in the main absorption intensity was observed among the three fractions. However, upon close comparison of the spectra, small differences are observed.

The broad absorption peak observed at around 3434-3420 cm^−1^ indicated the presence of intensive hydroxyl groups (O-H) stretching frequency confirming the polysaccharide nature of the material [58]. The C–H stretching vibration gives signals between 2928 and 2850 cm^−1^ [59]. The peak observed around 2366 cm^−1^ is attributed to O-H bond groups, which could be explained by their carbohydrate nature.

The absorption peaks at 1634 cm^−1^, 1628 cm^−1^, and 1626 cm^−1^ were due to the stretch vibration of carboxyl group (C = O) [60]. The absorption at 1404 and 1406 cm^−1^ was due to the symmetric stretching of –COO.

There were peaks near 1000-1200 cm^−1^, indicating that the polysaccharide contained *α*-pyranose. Indeed, the carbohydrates show high absorbencies in this region, which is within the so-called fingerprint region, where the position and the intensity of the bands are specific for every polysaccharide, allowing its possible identification [61].

The intense peaks at 1094 cm^−1^, 1074 cm^−1^, and 1048 cm^−1^ were attributed to the vibration of the glycosidic linkage C-O-C of glucose [60].

Along with these peaks, more characteristic peaks at 870 and 804 cm^–1^ region were also detected indicating that the EPS contained both *α* and *β*-type glycosidic linkages between sugar monomers [62]. These peaks are absent in the EPS fraction extracted from* Ln. mesenteroide*s B3.

The weak adoption band at 534-538 cm^−1^, absent in the EPS fraction purified from* Ln. mesenteroide*s B3, was indicative of glycosidic linkage peak for polysaccharide.

## 4. Conclusion

In this work, ultrafiltration and ethanol precipitation were used for the purification of EPSs produced by three Lactic Acid Bacteria strains isolated from different Tunisian biotopes. Results confirmed that EPS recovery by ultrafiltration was significantly higher than ethanol precipitation for* Lc. lactis* SLT10 and* Lb. plantarum* C7. GC-MS and HPSEC analysis of EPSs showed that the three studied strains produce a heteropolysaccharides with low-molecular masses. Depending on purification method, the monomeric composition and molar ratios of the different EPS fractions are affected.

## Figures and Tables

**Table 1:** Reducing sugars contents (%) of different exopolysaccharides fractions.

**Strains**	**EPS-ETOH**	**EPS-UF**
*Lc. Lactis* SLT10	28.06±0.041	33.63±1.38
*Lb. plantarum* C7	19.82±0.305	26.54±0.905
*Ln. mesenteroides *B3	22.07±0.019	22.044±0.366

**Table 2:** Proteins contents (%) of different exopolysaccharides fractions.

**Strains**	**EPS-ETOH**	**EPS-UF**
*Lc. Lactis* SLT10	1.008±0.049	1.284±0.048
*Lb. plantarum* C7	0.928±0.036	0.414±0.027
*Ln. mesenteroides *B3	0.925±0.033	0.808±0.067

**Table 3:** Sugar composition and molar ratio of exopolysaccharides isolated from the three studied strains (SLT10, C7, and B3).

**Strains**	**Monomers**	**Molar ratio**		**Mol (**%**)**	
		***EPS-ETOH***	***EPS-UF***	***EPS-ETOH***	***EPS-UF***
*Lc. Lactococcus* SLT10	Glucose	0.47±0.033	0.58±0.0001	1.97±0.008	8.46±0.35
	Mannose	1	1	4.15±0.27	14.59±0.6
	Rhamnose	-	0.18±0.002	-	2.48±0.13
	Total			6.13±0.264	25.55±1.09

*Lb. plantarum* C7	Glucose	0.607±0.162	4.07±0.82	3.34±1.31	13.36±8.11
	Mannose	1	1	6.01 ± 3.77	8.98±7.73
	Total			9.35 ± 5.09	22.34±2.73

*Ln. mesenteroides* B3	Glucose	0.61± 0.02	3.48± 0.02	1.97± 0.167	13.35± .09
	Mannose	1	1	3.21 ±0.38	3.85 ±0.62
	Total			5.19 ±0.54	17.20 ±2.72

**Table 4:** Molecular weight (Mw), molecular number (Mn), and polydispersity index of EPSs extracted from the three studied stains.

**Purification method**	**Strains**	**Mw (kDa)**	**Mn (kDa)**	**Polydispersity Index (Mw/Mn)**	**Mass recovery (**%**)**
*EPS-ETOH*	*Lc. lactis* SLT10	62.4±2.94	19.3±2.07	3.231±0.47	37.3
	*Lb. plantarum* C7	33.7±2.89	8.5±3.44	3.968±1.64	46.0
	*Ln. mesenteroides *B3	-	-	-	-

*EPS-UF*	*Lc. lactis* SLT10	51.5±1.75	12.3±1.38	4.178±0.49	54.6
	*Lb. plantarum* C7	11.6±1.83	2.1±0.95	5.586±2.66	53.5
	*Ln. mesenteroides *B3	18.6±2.21	8.4±1.52	2.204±0.407	46.1

## Data Availability

The data used to support the findings of this study are available from the corresponding author upon request.
